# Characterization of the Volatile Profiles of Insect Flours by (HS)-SPME/GC-MS: A Preliminary Study

**DOI:** 10.3390/molecules28073075

**Published:** 2023-03-30

**Authors:** Samantha Reale, Alessandra Biancolillo, Martina Foschi, Angelo Antonio D’Archivio

**Affiliations:** Dipartimento di Scienze Fisiche e Chimiche, Università degli Studi dell’Aquila, Via Vetoio, 67100 L’Aquila, Italy

**Keywords:** HS-SPME/GC-MS, insect food, edible insect flour: edible insect flavor, *Acheta domesticus*, *Alphitobius diaperinus*, *Tenebrio molitor*

## Abstract

The growing world population, combined with scarcities of agricultural land, water, forest, fisheries, and biodiversity resources, makes it necessary to search for alternative sources of nutrients. For this reason, in recent years, edible insects have been introduced into the diet, even in areas where entomophagy is not traditional. In light of this, the present study aims at characterizing the aromatic profile of three edible insects flours: cricket (*Acheta domesticus*, CP), buffalo worm (*Alphitobius diaperinus*, BW), and mealworm (*Tenebrio molitor*, MW). This goal has been achieved by means of an (HS)-SPME/GC-MS strategy. 67 compounds have been tentatively identified; of these, 27 are present only in the CP and BW flours, while 10 are common in all three flours. The compound with the highest peak’s relative area in gas chromatograms of CP and BW flours is hexadecanoic acid, while in MW it is 1-heptylpyrrolidin-2-one. In general, we have observed that CP and BW flours have 37 compounds in common, and their volatile compositions along with their profiles are more similar to each other than to MW profile.

## 1. Introduction

The ever-growing world population inevitably forces an increased food (and even feed) production from available agro-ecosystems, resulting in dramatic pressure on the environment. Scarcities of agricultural land, water, forest, fisheries, and biodiversity resources, as well as nutrients and non-renewable energy, are foreseen and need to be faced.

One possible answer for nutrient scarcity could be the introduction of insect-based food in the daily diet of Western populations, since edible insects are a potential reliable source of high quality protein (which contains all eight essential amino acids), fats, carbohydrates, and various vitamins and minerals with a very low environmental impact [1].

Regarding the environmental impact, insects are extremely sustainable, as their breeding is not connected to the availability of land. Moreover, insects are cold-blooded animals (taxonomically grouped into several families within the class Insecta) and their carbon dioxide and other greenhouse gas emissions are much lower than those from warm blooded animals such as farm animals. In this context, the requirements for water and nutrients are also reduced. Furthermore insects’ protein bioconversion rate is also considerably higher than that of farm animals [2].

Additionally, many insects also feed on organic waste, thus solving another very topical problem, namely that of food waste: the United Nations estimates that, around the world, about one third of food destined for human consumption is thrown away [3].

There are nearly 1900 insect species reported for human consumption and more than 2 billion people use insects as food. Edible insects are mostly consumed in Asia and Central America, while in the Western world, entomophagy remains rare or even a taboo [4].

Although health and environmental issues are powerful motivations to entomophagy, it has been suggested that people are more likely to be persuaded by a positive eating experience [5]. In this context, besides considering the nutritional and safety aspects of edible insects, it would be extremely important to also take into consideration the appearance and flavor profile of insect-based foods.

Regarding the appearance, since in Western countries the acceptance of insect consumption is very low, the main form of insect consumption is as an ingredient; typically, a powder obtained from grinding dehydrated insect or protein extracts is added to food products such as cookies, snack, bars and protein shakes. In fact, incorporating insects in a non-recognizable form, such as a powder or a flour, reduces the negative perception of eating insects. In fact, it has been reported that the low acceptability of insects as food is mostly associated with emotional factors such as disgust, as well as unusual tastes and textures, and thus the use of edible insects in a unrecognizable form as an ingredient could be a potential means to increase edible insect acceptance [6]. For instance, European and American consumers have shown a liking towards food products containing insects in the form of protein flours, thus in an unrecognizable form [7].

The flavor of edible insects depends on the insect species, their stage of development, and for insect-based food also on the method of drying and preparation. Even though the edible insect flavor is generally described as tasty [8], it is still not sufficient to motivate consumers who are hostile to insect consumption. Undoubtedly, the final insect-based food’s flavor, either positively or negatively, greatly affects consumers’ attitude. Consequently, since the sensory properties are essential when commercializing new food products, understanding the complex taste and flavor of edible insects and the impact of processing methods on the organoleptic characteristics is crucial to improve consumer acceptance of insect-derived foods.

Most of the studies on edible insects are focused on their nutritional composition, while there is still a lack of knowledge on organoleptic properties of these novel foods. This gap needs to be undertaken, since it is known that the analysis of the volatilome of a food product provides an important indication of the safety and the authenticity of a food product [9].

One of the reasons is definitely the extreme variability of the volatile compounds among species, stage of development and, above all, edible insect processing methods.

A very recent comprehensive review on volatile compounds found in edible insect [10] reports about twenty papers dealing with characterization of volatiles in edible insects, and virtually every paper discusses a different insect species or development stage, or a different sample form (whole insect, part of body insect, powder, protein extract, food containing edible insect, etc) or even a different edible insect processing method (for instance, different drying methods or temperatures and diverse cooking methods). The most used analytical technique to evaluate the flavor of edible insects is headspace solid-phase microextraction, (HS)-SPME combined with gas-chromatography coupled to mass spectrometry detection (GC-MS) [10]. SPME either in the head space mode or in other modes, is extremely widespread and increasingly used in the characterization of a wide variety of food matrices [11,12,13,14,15,16,17].

In the present study, we describe the volatile profile obtained by (HS)-SPME/GC-MS of flours of three (out of the only four) edible insects approved as novel foods in the European Union (see Section 4.1.1. for details), namely the house cricket (*Acheta domesticus*), buffalo worm larvae (also known as lesser mealworm, *Alphitobius diaperinus*) and mealworm larvae (*Tenebrio molitor*).

Regarding these three insect species’ volatile profiles studied by SPME, Khatun et al. characterized, also on the basis of the volatile profiles obtained by SPME, the house cricket powders obtained in the laboratory under three different processing methods [18]. Grossmann et al. described the flavoring potential of house crickets and mealworm protein hydrolysates [19]. Another study on mealworm deals with SPME characterization of the larvae affected by different roasting processes [20]

However, to the best of our knowledge, there are no studies on the characterization of the volatile component of commercial flours of these three edible insects by (HS)-SPME/GC-MS.

## 2. Results

The insect flours deriving from adult crickets (*Acheta domesticus*—CP), buffalo worm larvae (*Alphitobius diaperinus*—BW) and meal worm larvae (*Tenebrio molitor*—MW) have been analysed by HS-SPME/GC-MS under the same experimental conditions (reported in Section 4). The 67 volatile compounds tentatively identified in the GC-MS chromatograms along with their retention time (RT), the experimental and literature retention indices and the mean relative (%) area in the analysed samples are reported in Table 1.

As an overall consideration, it must be noted that the gas chromatograms (Figure S1) have low intensities for all three flours analysed here. In fact, the highest peak in the MW chromatograms barely surpasses 100 kcounts, while in the BW and CP ones the highest peaks are around 200 kcounts. Furthermore, most of the identified compounds have a relative intensity below 2%.

In all three flours analysed, we have detected and tentatively identified a total of 67 compounds, 27 of which are present only in the CP and BW flours, while only 10 (in italic in Table 1) have been found in all three flours. Since the CP and BW flours have 37 compounds in common, their volatile compositions along with their profiles are more similar to each other than to MW profile. It is worth emphasizing that the CP and BW samples were bought directly as flours, while in the case of MW we bought the larvae which were hand-grinded with a mortar before analysis.

The compound with the highest peak’s relative area in CP and BW flours is hexadecanoic acid (#61), which account for around 15% in CP flour and around 12% in the BW one. Conversely, this compound has not been detected at all in the MW flour. Remarkably, only two (#11 and #56) out of the six identified fatty acids were detected in MW flour; moreover, hexanoic acid (#11), the fatty acid with the shortest chain detected here, is present only in the MW flour (even if in trace amounts, around 1%). Together, the six fatty acids detected account for around 22% and 18% in CP and BW flours, respectively, and less than 2% in MW flour.

The compound with the highest peak’s relative area in the MW flour volatile profile is 1-heptylpyrrolidin-2-one #45, which accounts for about 18% in MW flour and less than 1% in both CP and BW flours. Noticeably, the five 1-alkylpyrrolidin-2-ones represent by far the compounds with the highest peak’s relative areas present in the MW flour’s volatile profile, where their overall amount is about 36%. On the contrary, the 1-alkylpyrrolidin-2-ones accounts for only a total relative area of 0.4% and 3% in CP and BW flours, respectively.

27 out of the 67 detected compounds listed in Table 1 can be considered as Maillard reaction products (MRPs) [21,22,23,24] and are highlighted by “a” apex in Table 1. Four out of these 27 MRPs were detected in all flours, namely two piperidines (#6 and #42) and two pyrrolidinones (#36 and #45). The cumulative area of MRPs in CP and BW flours is about 43%, while in MW flour it reaches around 49%.

Among the MRPs, we found 5 alkyl pyrazines (#7, #9, #21, #32 and #35) which account for around 24% of the total area in CP flour and around 14% in BW flour, while they are completely absent in MW flour. Pyrazines, which possess roasted, toasted, nutty, coffee-like, or cocoa-like odor notes, are the most abundant heterocyclic compounds among the volatile MRPs [25]. Interestingly. 2,5-dimethylpyrazine (#9) was found by Khatun at al. [18] in the HS-SPME GC MS profile of an oven-dried *A. domesticus* sample. Moreover, several different pyrazines were found in the HS-SPME GC MS profile of *T. molitor* larvae affected by roasting processes [20].

On the other hand, the five 1-alkylpyrrolidin-2-ones (#36, #40, #45, #55 and #58), as mentioned above, reach overall around 36% of the total area in MW flour, while contributing to about 0.4% and 3% in the chromatograms of CP and BW flour, respectively. Pyrrolidin-2-ones (mainly with a roasted cereal aroma), besides being Maillard products deriving from γ-aminobutyric acid [22,26], have been found in dried vegetables food where they are supposed to take origin from γ-aminobutyric acid ring closure to the lactam under dehydrating conditions or from monosodium glutamate upon thermal treatment [27].

Also, the seven compounds with a piperidine moiety (#4, #6, #8, #14, #19, #42 and #51) could be considered as MRPs, since it was demonstrated that lysine can generate piperidine in the presence of sugars [28], and piperidines are generally listed among the typical *N*-heterocyclic compounds’ flavor deriving from Maillard reactions [21]. Furthermore, piperidine is a reactive amine capable of undergoing Maillard type interactions, for example reacting with furfural to give (1-piperidinyl)-furfural (#51) but also yellow pigments, as demonstrated by Hofmann [29]. The cumulative relative amount of these seven piperidine MRPs is around 6% and 3% in CP and MW flours, respectively, while is around 17% in BW flour.

Two *N*-valeryl-l-proline-alkyl esters (#60 and #63) were detected only in CP and BW samples where they account for an overall relative area of around 2% in CP flour and around 15% in BW. *N*-valeryl-l-proline-alkyl esters have been identified in waste chicken feathers [30] but also in four scorpion species’ telsons [31] and in an African fermented condiment [32].

Besides, we identified five methlylketones which are reasonably produced by lipid oxidation [33]. The five tentatively identified methylketones account for only around 2% of the total area for BW flour and around 10% for MW flour, while in CP flour they reach an overall relative area of more than 20% and the heptadecane-2-one (#59) is the third most aboundant compound in CP flour with a peak’s relative area of about 10%.

γ-Heptalactone (#26) and γ-nonalactone (#37) were detected only in MW flour. Both of these γ-lactones have been described as important flavor compounds; in particular, the former possess a fatty milky coconut odor while the latter gives a fruity aroma [34]. We also detected γ-palmitolactone (#64) in all three flours.

## 3. Discussion

The comparison of the three flours’ volatile profiles shows a great similarity, in terms of compounds detected, between those of CP and BW flours compared to the volatile profile of BW. In fact, for CP and BW flours, we detected the same 37 compounds; consequently, the differences between the CP and BW flours’ volatile profiles depend only on the different relative abundances of these compounds. Conversely, in the case of MW flour, only 10 compounds out of the 38 volatiles identified for this flour are in common with the other two flours; thus, in this case, the volatile profile of MW flour appears completely different, mainly in qualitative terms.

What mostly affects the volatile profile seems not to be the insect phylogenetic classification (all the three insect belong to the Arthropoda phylum, but rather BW and MW belong to the same family, Tenebrionidae, while CP belongs to that of Gryllidae) nor its development stadium (CP flour is obtained from adult crickets, while BW and MW flours from larvae), but probably the flour production process. In fact, CP and BW were purchased as flours, while MW has been bought as whole larvae and hand grinded.

The presence of a high number and relative amount of MRPs in the three flour samples (17 MRPs in CP and BW flours and 12 in MW flour with a cumulative area of about 43% in both CP and BW flours and around 49% in MW flour) could, at first sight, be considered an experimental artifact due to the relatively high temperature (130 °C) and long time (1 h 45 min) at which the insect flours were exposed during the SPME analysis, which, in principle, could simulate a sort of cooking process. However, this hypothesis must be discarded since Maillard reaction products are also reported in volatile profiles of proteinaceous flours from legumes obtained by (HS)-SPME/GC-MS at 65 °C [35] or by solvent-assisted flavor extraction [36]. Furthermore, Khatun et al. [18] reported the presence of MRPs in the volatile profile of house cricket powder oven dried at 65 °C.

Interestingly, in a (HS)-SPME/GC-MS study on the characterization of volatiles deriving from the roasting process on *Tenebrio molitor* larvae (the same larvae from which we obtained MW flour), the authors found some pyrazines [20]. The fact that in the gas chromatogram of MW flours we did not detect pyrazines at all, while in the volatile profiles of CP and BW flours the five detected alkyl pyrazines account for 25% and 14%, respectively, is another indication that these compounds (and also the other MRPs) are not experimental artifacts. The above compounds, rather, derive from the flour production processes, plausibly exposing the insects to higher temperatures and stronger dehydrating conditions [37] than those experimented by MW flour, which, as already said, was obtained by hand grinding the intact *Tenebrio molitor* larvae.

## 4. Materials and Methods

### 4.1. Samples

- CP: flour from *Acheta domesticus* “Cricket Powder”, purchased from GRIG Distribuce s.r.o. Czech Republic;
- BW: flour from *Alphitobius diaperinus*, purchased from isaac nutrition Gesellschaft mit beschränkter Haftung. Germany;
- MW: dried larvae from *Tenebrio molitor*, purchased from Sahawa, Germany. The larvae were hand grinded in a mortar to obtain a powder similar to the CP and BW flours.

#### 4.1.1. EU Samples’ Authorization as Novel Food

The larval form of the insect species *Tenebrio molitor*—also known as the (yellow) mealworm—was authorized as novel food on 3 May 2021 (reg. EU 2021/882 and reg. EU 2022/169), and was the first insect-based novel food authorized in the EU.

The adult form of house cricket (*Acheta domesticus*) was authorized as novel food on 3 March 2022 (reg. EU 2022/188).

The larval form of *Alphitobius diaperinus* (also known as buffalo worm and lesser mealworm) was authorized by the EFSA on 4 July 2022 (EFSA-Q-2018-00282 ON-7325) and the EU executive regulation has not yet been issued. (Register of Questions European Food Safety Authority. Available online: https://open.efsa.europa.eu/questions; accessed on 16 November 2022)

### 4.2. Headspace Solid-Phase Micro-Extraction (HS-SPME)

The SPME fiber used in this work was coated with divinylbenzene/carboxen/polydimethylsiloxane (DVB/CAR/PDMS), 50/30 μm thickness (Supelco, Bellefonte, PA, USA).

About 100 mg of flour was placed in a 4 mL vial equipped with the Mininert cap and kept at 130 °C in an oil bath for 1 h without stirring. The fiber was introduced into the vial and exposed for 45 min to the head space of the sample. After insertion of the fiber into the injection port of the GC apparatus, desorption of the extracted volatiles took place at 250 °C for 2 min. After each analysis, the fiber was kept in the GC injection port at 270 °C for 5 min, and after that a blank run was recorded to check the cleaning of the sorbent.

For each sample, the analysis was done in triplicate.

### 4.3. Gas Chromatography-Mass Spectrometry (GC-MS) Analysis

All the analyses were carried out on a Saturn 2000 GC-MS instrument (Varian, Inc. Palo Alto, CA, USA). The GC apparatus was equipped with a 1078 split/splitless injector with a SPME liner inside. All injections were performed in split mode with a 10:1 split ratio.

A Varian FactorFour™ VF5-ms capillary column (5% phenyl-95% polymethylsiloxane, 30 m × 0.25 mm × 0.25 μm film thickness) was used and the carrier gas was helium 5.5 IP, supplied at a flow rate of 1.1 mL/min. GC separation was achieved using the following column oven temperature program: initial temperature 50 °C for 2 min, then increasing to to 100 °C at 30 °C/min, then to 250 °C at a rate of 6 °C/min, at the end the temperature of 280 °C was reached at 65 °C/min and held for 2 min. The EI-ion trap mass spectrometer operated at the following conditions: ionization energy 70 eV; 40–600 *m/z* range; 1 s scan cycle time; transfer line temperature 170 °C; manifold temperature 110 °C; ion trap temperature 150 °C. The compounds were identified by comparison of their linear retention indices calculated using a linear alkane mixture (Retention Index Standard-aliphatic C_7_–C_40_ hydrocarbons dissolved in hexane—from Sigma-Aldrich, Saint Louis, MO, USA) and their mass spectra with those reported in standard libraries [38].

## 5. Conclusions

In this preliminary study, we described the volatile profiles of three edible insect flours, namely flours from adult crickets (*Acheta domesticus*), buffalo worm larvae (*Alphitobius diaperinus*) and meal worm larvae (*Tenebrio molitor*—MW), all approved as novel food by the European Union.

The volatile profiles obtained by (HS)-SPME/GC-MS from each of the three flours under investigation appear quite peculiar and seem to depend both on insect species and flour processing.

On the basis of this preliminary evidence, SPME can be regarded as an effective and promising tool for future applications aimed at describing and discriminating flour of different insects and/or obtained by different technological processes.

The potential effectiveness of insect flours’ volatile profiles as a fingerprint for insect species supports the upcoming application of (HS)-SPME/GC-MS to assess the authenticity of these products and detect possible food frauds deriving from their adulteration with lower value flours (either from other insect species or deriving from legumes).

## Figures and Tables

**Table 1:** Volatile profiles of insect flours: peak number (#); observed retention time (RT (min)), assigned chemical structure Experimental and literature retention indices and mean relative (%) peak area in the GC-MS chromatograms (calculated as the percentage of each compound’s peak area on the sum of the peak areas of the 67 compounds tentatively identified) with related standard error (se) for each flour: CP: flour from cricket (*Acheta domesticus*); BW: flour from buffalo worm larvae (*Alphitobius diaperinus*); MW: flour from meal worm larvae (*Tenebrio molitor*).

			Retention Index		CP (n = 3)	BW (n = 3)	MW (n = 3)
**#**	RT (min)	Compound *	Exp ^1^	Lit ^2^	Mean A% ± se	Mean A% ± se	Mean A% ± se
1	2.399	*3-ethylpentane*	684	686 ± 1	2.11 ± 0.90	4.71 ± 0.31	16.70 ± 6.05
2	2.775	(methyldisulfanyl)methane	743	746 ± 6	0.61 ± 0.31	1.14 ± 0.19	n.d.
3	2.891	pentan-1-ol	761	765 ± 4	n.d.	n.d.	1.58 ± 0.13
4	2.930	piperidine ^a^	767	764 ± 2	0.04 ± 0.04	0.56 ± 0.03	n.d.
5	3.090	hexan-2-one	792	790 ± 3	n.d.	n.d.	3.81 ± 0.27
6	3.395	*3-methylpiperidine* ^a^	825	823 ± 0	0.26 ± 0.18	2.91 ± 0.21	0.031 ± 0.007
7	3.493	2-methylpyrazine ^a^	834	831 ± 7	11.52 ± 2.04	2.77 ± 0.47	n.d.
8	3.571	2,3-dimethylpiperidine ^a^	842	n/a	0.13 ± 0.02	0.53 ± 0.05	n.d.
9	4.319	2,5-dimethylpyrazine ^a^	914	917 ± 4	2.77 ± 0.43	3.87 ± 0.39	n.d.
10	4.976	dimethyltrisulfane	977	970 ± 7	1.58 ± 0.18	0.99 ± 0.18	n.d.
11	4.980	hexanoic acid	978	990 ± 16	n.d.	n.d.	1.17 ± 0.25
12	5.035	oct-1-en-3-ol	983	980 ± 2	n.d.	n.d.	1.38 ± 0.07
13	5.130	2-pentylfuran ^a^	992	993 ± 2	n.d.	n.d.	2.31 ± 0.15
14	5.450	2-ethylpiperidine ^a^	1020	n/a	3.83 ± 0.76	9.27 ± 0.34	n.d.
15	5.530	*2-methyl-N-(2-methylbutyl)butan-1-imine*	1026	1025 ± 0	0.54 ± 0.29	3.55 ± 0.21	0.20 ± 0.03
16	5.609	2-ethylhex-2-enal	1031	999 ± 30	n.d.	n.d.	1.36 ± 0.17
17	5.724	3-methyl-*N*-(3-methylbutyl)butan-1-imine	1042	1047 ± n/a	0.59 ± 0.27	3.05 ± 0.36	n.d.
18	5.950	2-acetylpyrrole ^a^	1060	1064 ± 5	3.81 ± 0.72	2.93 ± 0.67	n.d.
19	6.14	piperidin-4-ylmethanamine ^a^	1076	n/a	n.d.	n.d.	2.01 ± 0.30
20	6.180	octa-3,5-dien-2-one	1079	1073 ± 7	n.d.	n.d.	0.73 ± 0.10
21	6.260	3-ethyl-2,5-dimethylpyrazine ^a^	1085	1082 ± 3	7.87 ± 0.73	2.94 ± 0.52	n.d.
22	6.323	nonan-2-one	1090	1092 ± 2	4.66 ± 0.83	0.72 ± 0.03	2.10 ± 0.09
23	6.380	hygrine (1-[(2*R*)-1-methylpyrrolidin-2-yl]propan-2-one) ^§ a^	1095	1093 ± 2	n.d.	n.d.	2.90 ± 0.31
24	6.410	hexanamide	1098	n/a	n.d.	n.d.	1.40 ± 0.23
25	6.520	4-methylpyrimidin-2-amine ^a^	1105	n/a	0.28 ± 0.05	2.54 ± 0.54	n.d.
26	7.280	γ-heptalactone (5-propyloxolan-2-one) ^§^	1056	1159 ± 4	n.d.	n.d.	2.12 ± 0.71
27	7.650	piperidin-2-one ^a^	1781	1174 ± n/a	6.83 ± 1.19	0.98 ± 0.09	n.d.
28	7.806	decan-2-one	1191	1193 ± 2	7.23 ± 1.42	0.59 ± 0.07	2.91 ± 0.23
29	7.945	1-pentylpyrrole ^a^	1200	n/a	n.d.	n.d.	2.08 ± 0.26
30	8.041	1-but-1-enylpyrrolidine ^a^	1206	n/a	0.56 ± 0.11	1.03 ± 0.19	n.d.
31	8.401	(methyltetrasulfanyl)methane	1227	1234 ± 11	1.10 ± 0.17	1.17 ± 0.09	n.d.
32	8.882	2-methyl-6-(3-methylbutyl)pyrazine ^a^	1250	1249 ± 4	0.36 ± 0.05	0.80 ± 0.16	n.d.
33	9.068	2-phenylacetic acid	1266	1262 ± 5	0.13 ± 0.09	3.29 ± 0.26	n.d.
34	9.603	1H-indole	1297	1295 ± 7	n.d.	n.d.	1.64 ± 0.14
35	9.904	2,5-dimethyl-3-(3-methylbutyl)pyrazine ^a^	1313	1315 ± 7	1.98 ± 0.38	3.70 ± 0.81	n.d.
36	10.761	*1-butylpyrrolidin-2-one* ^a^	1360	n/a	0.24 ± 0.02	2.46 ± 0.53	6.75 ± 0.44
37	10.945	γ-nonalactone (5-pentyloxolan-2-one) ^§^	1369	1363 ± 5	n.d.	n.d.	2.41 ± 0.33
38	11.265	α-ionol ((E)-4-(2,6,6-trimethylcyclohex-2-en-1-yl)but-3-en-2-ol) ^§^	1387	1390 ± 3	n.d.	n.d.	0.06 ± 0.02
39	11.302	2-butyloct-2-enal	1389	1378 ± 10	n.d.	n.d.	0.30 ± 0.04
40	11.393	1-pentylpyrrolidin-2-one ^a^	1394	n/a	n.d.	n.d.	2.81 ± 0.29
41	11.684	pyridine-4-carboxamide	1409	1408 ± n/a	n.d.	n.d.	1.81 ± 0.16
42	12.531	*2-benzylpiperidine* ^a^	1453	n/a	0.39 ± 0.03	2.17 ± 0.08	0.82 ± 0.11
43	12.893	3-phenylpyridine ^a^	1472	1467 ± 2	0.54 ± 0.36	0.61 ± 0.02	n.d.
44	13.32	5,6-dihydro-6-pentyl-2H-pyran-2-one	1495	1501 ± 19	n.d.	n.d.	0.45 ± 0.07
45	13.451	*1-heptylpyrrolidin-2-one* ^a^	1502	n/a	0.16 ± 0.07	0.85 ± 0.03	18.51 ± 1.94
46	13.590	2-hexyldecan-1-ol	1509	1504 ± n/a	n.d.	n.d.	0.87 ± 0.15
47	13.797	2,5-diethoxyaniline	1520	n/a	n.d.	n.d.	1.67 ± 0.14
48	14.253	1-pyrrolidin-1-yldecan-1-one ^a^	1554	n/a	n.d.	n.d.	1.45 ± 0.10
49	14.521	naphthalen-2-amine	1559	1555 ± 7	2.40 ± 0.72	4.71 ± 0.55	n.d.
50	15.639	1-pyrrolidin-1-yldodecan-1-one ^a^	1618	n/a	n.d.	n.d.	1.41 ± 0.11
51	16.729	(1-piperidinyl)-furfural (5-piperidin-1-ylfuran-2-carbaldehyde) ^§ a^	1678	n/a	1.24 ± 0.32	1.87 ± 0.60	n.d.
52	16.750	tetradecan-1-ol	1679	1676 ± 4	n.d.	n.d.	1.03 ± 0.21
53	16.888	(Z)-pentadec-11-enal	1686	1694 ± 8	n.d.	n.d.	1.64 ± 0.31
54	17.231	pentadecan-2-one	1705	1698 ± 4	n.d.	n.d.	1.58 ± 0.02
55	17.495	1-benzylpyrrolidin-2-one ^a^	1720	n/a	n.d.	n.d.	4.04 ± 0.32
56	18.442	*tetradecanoic acid*	1774	1768 ± 5	0.61 ± 0.20	0.48 ± 0.04	0.42 ± 0.05
57	18.75	octadec-1-ene	1791	1793 ± 1	n.d.	n.d.	0.72 ± 0.16
58	19.896	1-decylpyrrolidin-2-one ^a^	1858	n/a	n.d.	n.d.	3.53 ± 0.30
59	20.603	heptadecan-2-one	1900	1902 ± 7	9.75 ± 1.52	0.61 ± 0.03	n.d.
60	21.364	N-valeryl-l-proline-pentyl ester (hexadecyl 1-pentanoylpyrrolidine-2-carboxylate) ^§^	1947	n/a	0.95 ± 0.35	6.61 ± 0.55	n.d.
61	21.661	hexadecanoic acid	1966	1968 ± 7	14.66 ± 6.25	12.21 ± 2.64	n.d.
62	22.725	geranyl linalool	2031	2034 ± n/a	n.d.	n.d.	0.87 ± 0.05
63	22.750	N-valeryl-l-proline-hexyl ester (hexadecyl 1-hexanoylpyrrolidine-2-carboxylate) ^§^	2034	n/a	1.47 ± 0.51	7.94 ± 0.75	n.d.
64	23.820	*γ-palmitolactone (5-dodecyloxolan-2-one) ^§^*	2103	2105 ± 1	1.93 ± 0.42	0.28 ± 0.01	0.41 ± 0.03
65	24.246	(9*Z*,12*Z*)-octadeca-9,12-dienoic acid	2132	2133 ± 12	2.08 ± 0.65	2.09 ± 1.00	n.d.
66	24.342	(Z)-octadec-9-enoic acid	2138	2141 ± 11	2.41 ± 0.99	2.71 ± 0.97	n.d.
67	24.21	octadecanoic acid	2164	2172 ± 7	2.34 ± 0.51	0.34 ± 0.13	n.d.

* Compound names according to IUPAC recommended nomenclature. ^a^ Compound deriving from Maillard reaction. ^§^ Recommended IUPAC name in parenthesis. Compounds in italic are those detected in all three flours under investigation. ^1^ Exp: experimental retention indices calculated using a C_7_–C_40_ linear alkane mixture. ^2^ Lit.: semistandard non-polar retention indices reported in the NIST14 mass spectra database. n.d.: not detected. n/a: not available.

## Data Availability

Not applicable.

## Supplementary Materials

The following supporting information can be downloaded at: https://www.mdpi.com/article/10.3390/molecules28073075/s1, Figure S1: Gas chromatograms of: (a) Buffalo Worm larvae flour; (b) Cricket flour; (c) Meal Worm larvae flour.
