# Supplementary material for: Characterization of the Volatile Profiles of Insect Flours by (HS)-SPME/GC-MS: A Preliminary Study
Source: Molecules. 2023 Mar 30;28(7):3075. doi: 10.3390/molecules28073075 (PMC10095912; doi:10.3390/molecules28073075)
Supplement: Supplementary file 1 [file molecules-28-03075-s001.zip › molecules-2147503-supplementary.pdf]

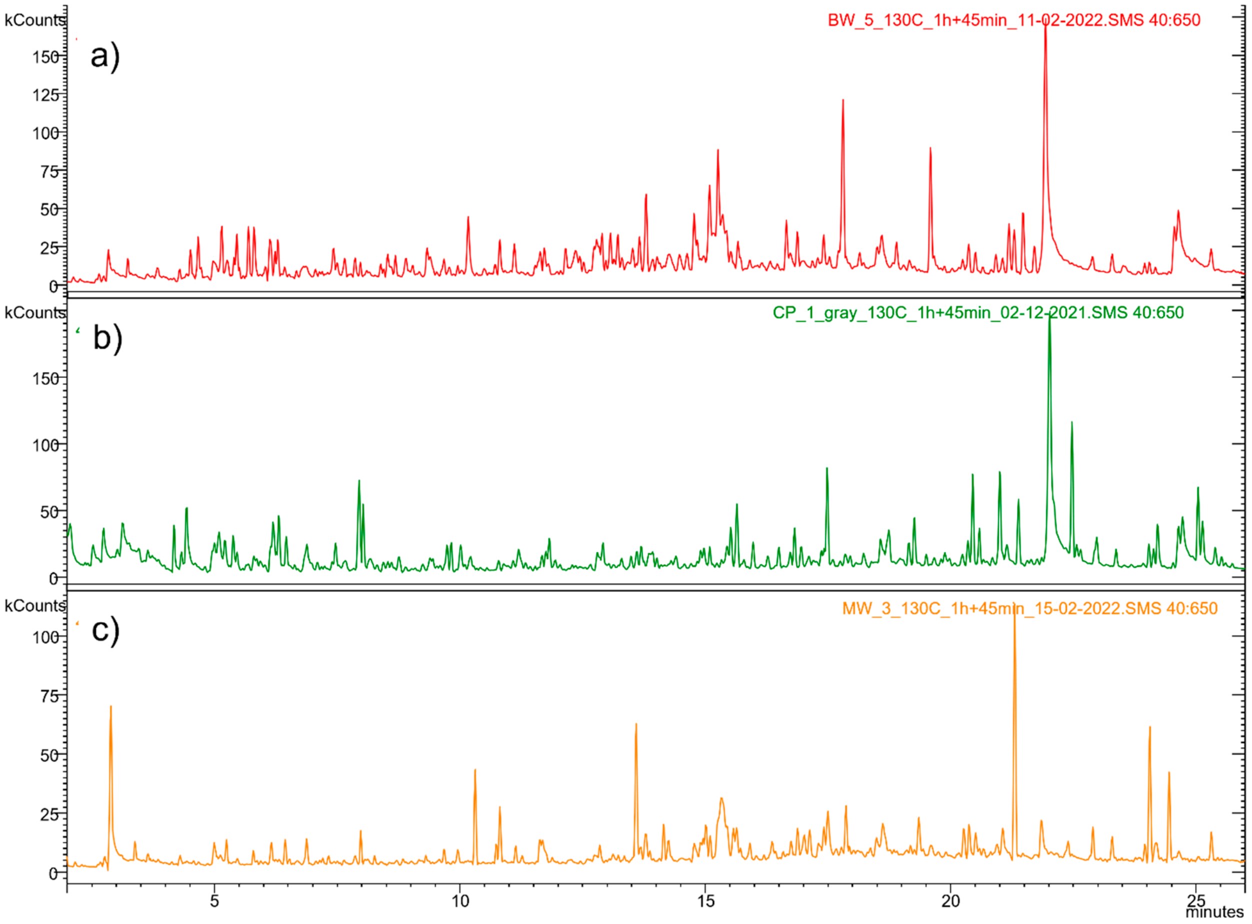

Figure S1: Gas chromatograms of: a) Buffalo Worm larvae flour; b) Cricket flour; c) Meal Worm larvae flour
